# Cryo-EM structure of the prefusion state of canine distemper virus fusion protein ectodomain

**DOI:** 10.1016/j.yjsbx.2020.100021

**Published:** 2020-02-29

**Authors:** David Kalbermatter, Neeta Shrestha, Flavio M. Gall, Marianne Wyss, Rainer Riedl, Philippe Plattet, Dimitrios Fotiadis

**Affiliations:** a Institute of Biochemistry and Molecular Medicine, University of Bern, Bern, Switzerland; b Division of Experimental and Clinical Research, Vetsuisse Faculty, University of Bern, Bern, Switzerland; c Division of Neurological Sciences, Vetsuisse Faculty, University of Bern, Bern, Switzerland; d Center of Organic and Medicinal Chemistry, Institute of Chemistry and Biotechnology, Zurich University of Applied Sciences ZHAW, Wädenswil, Switzerland

**Keywords:** CDV, canine distemper virus, cryo-EM, cryo-electron microscopy, FP, fusion peptide, MeV, measles virus, Canine distemper virus, Cryo-electron microscopy, Fusion protein, Morbillivirus cell entry, Single particle reconstruction

## Abstract

Measles virus (MeV) and canine distemper virus (CDV), two members of the *Morbillivirus* genus, are still causing important global diseases of humans and animals, respectively. To enter target cells, morbilliviruses rely on an envelope-anchored machinery, which is composed of two interacting glycoproteins: a tetrameric receptor binding (H) protein and a trimeric fusion (F) protein. To execute membrane fusion, the F protein initially adopts a metastable, prefusion state that refolds into a highly stable postfusion conformation as the result of a finely coordinated activation process mediated by the H protein. Here, we employed cryo-electron microscopy (cryo-EM) and single particle reconstruction to elucidate the structure of the prefusion state of the CDV F protein ectodomain (solF) at 4.3 Å resolution. Stabilization of the prefusion solF trimer was achieved by fusing the GCNt trimerization sequence at the C-terminal protein region, and expressing and purifying the recombinant protein in the presence of a morbilliviral fusion inhibitor class compound. The three-dimensional cryo-EM map of prefusion CDV solF in complex with the inhibitor clearly shows density for the ligand at the protein binding site suggesting common mechanisms of membrane fusion activation and inhibition employed by different morbillivirus members.

## Highlights

• Expression and purification of prefusion CDV solF in presence of a fusion inhibitor.
• Elucidation of the CDV fusion protein ectodomain by cryo-EM.
• High structural similarity between MeV and CDV solF suggests common fusion mechanisms.

## Graphical abstract

**Figure:**
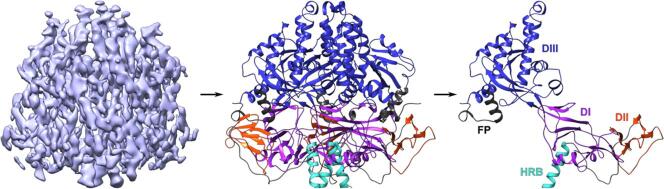

## 1 Introduction

Despite the availability of an efficient vaccine, measles virus (MeV) is still causing outbreaks worldwide that are associated with major childhood morbidity and mortality in developing countries (Simons et al., 2012). In most cases, MeV induces an acute disease, although fatal persistent infections of the central nervous system (CNS) may occasionally occur (Laksono et al., 2016, Perry and Halsey, 2004, Ferren et al., 2019). MeV is a pleomorphic enveloped, negative-sense, single-stranded RNA virus that belongs to the genus *Morbillivirus* of the family *Paramyxoviridae* (de Vries et al., 2015). Morbilliviruses also include canine distemper virus (CDV), which infects a broad range of terrestrial and aquatic animals, including endangered species, e.g., giant pandas (Fenget al., 2016, Jinet al., 2017). Noteworthy, despite an overall pathogenesis closely related to MeV, CDV induces much more frequently severe disorders in the central nervous system (Lempp et al., 2014).

The morbillivirus cell entry system relies on the concerted action of two glycoproteins anchored in the viral envelope: the receptor binding (H) protein and the fusion (F) protein. Initially, the H protein binds to a surface receptor (SLAM in immune cells or nectin-4 in epithelial cells), thereby determining the viral tropism. On the other hand, the putative receptor(s) of MeV and CDV expressed in brain cells remain(s) to be determined. Upon receptor binding, it is assumed that the H protein activates the F protein presumably through a series of tightly coordinated conformational changes. In turn, the fusion protein dramatically refolds to fuse the envelope of the virus with the host cell plasma membrane, which allows for the injection of the viral nucleocapsid (containing the genetic information) into the host cell cytoplasm (Navaratnarajah et al., 2020, Plattetet al., 2016, Plattet and Plemper, 2013).

Both, MeV and CDV F protomers are first synthesized as long inactive precursors, which must be cleaved by furin proteases residing within the Golgi apparatus to achieve trimeric fusion-competent, disulfide-linked, F1 and F2 subunits. Such primed trimeric F complexes are characterize by a high energy-containing state (i.e., metastable), which is commonly referred to as the prefusion conformation. F protomers contain well-characterized functional domains: an N-terminal signal peptide, N- and C-terminal heptad repeat (HRA and HRB) domains, a transmembrane region and a C-terminal cytosolic tail. Importantly, upon proteolytic cleavage, a new N-terminal hydrophobic region is generated, also known as the fusion peptide (FP) (Plattet et al., 2016). To fuse the viral envelope with the host cell plasma membrane, it is assumed that activated prefusion F complexes first refold into an intermediate state (referred to as the pre-hairpin intermediate (PHI) structure), which propels the FP towards the target membrane. Subsequently, PHI complexes undergo further structural rearrangements to ultimately achieve a highly-stable and low energy state (referred to as the postfusion conformation), which is associated with membrane merging. The postfusion conformation is characterized by a typical six-helix bundle (6HB) core domain that results from the assembly of three HRA and HRB domains (Brindleyet al., 2014, Plattetet al., 2016). Noteworthy, 6HB structures are reminiscent to all class I viral glycoproteins (e.g., influenza HA, Ebola GP or human immunodeficiency virus ENV GP41 proteins) in their postfusion conformation (Harrison, 2015, Kielian, 2014).

Recently, the crystal structure of the MeV F ectodomain in the prefusion state (MeV solF) was determined (Hashiguchi et al., 2018). The structure was closely related to previously elucidated prefusion forms of fusion glycoproteins of other paramyxoviruses and pneumoviruses. Such structures displayed “tree-like” shapes featuring a stalk domain supporting a large globular, or more oval, head region (Hashiguchiet al., 2018, McLellanet al., 2013, Welchet al., 2012, Wonget al., 2015, Xuet al., 2015, Yinet al., 2005). To achieve proper expression yield of MeV solF, Hashiguchi and colleagues employed a “locked-stalk” approach that is based on the introduction of supplementary stabilizing disulfide bridges within the stalk region (Hashiguchi et al., 2018); a strategy recently proven to be effective in case of stabilizing the prefusion state of the respiratory syncytial virus F protein (Stewart-Jones et al., 2015). Two structurally-independent inhibitors of the fusion process (AS-48 and FIP) were also co-crystallized with MeV solF prefusion constructs. Both inhibitors were demonstrated to dock on identical pocket microdomains, which localized at the structural transition between the head and the stalk domain with a stoichiometry of three ligands per trimeric complex (Hashiguchi et al., 2018).

Here, to stabilize soluble CDV F ectodomain (solF) in the prefusion state, we fused the trimeric GCNt peptide to the stalk domain, and expressed and purified solF in mammalian cells in the presence of 3G (Sunet al., 2006, Singethanet al., 2010), a derivative of AS-48 (Plemper et al., 2005). We recently reported that this strategy efficiently stabilizes prefusion solF (Kalbermatter et al., 2019). By employing cryo-electron microscopy (cryo-EM) and single particle reconstruction, we successfully determine the three-dimensional (3D) structure of prefusion CDV solF at 4.3 Å resolution.

## 2 Materials and methods

### 2.1 Expression and purification of CDV solF in the presence of 3G

The CDV solF construct (Ader et al., 2013) was codon-optimized and sent to a protein-expression core facility for protein production (LBTC, EPFL, Switzerland). Briefly, ~2·10^9^ of HEK293 cells grown in suspension were transfected with 3 mg of expression plasmid. Cells were grown in the presence of 75 µM 3G fusion inhibitor, which we synthetized as described previously (Sun et al., 2006). The supernatant (1 l) was harvested after seven days of expression at 37 °C. Then, 2 ml of hemagglutinin (HA) epitope-tag antibody-agarose conjugate (50% slurry, Pierce™) was filled into an Econo-Column® Chromatography Column, 1.0 × 5 cm (BioRad). The column was packed and first rinsed with buffer A (20 mM Tris-HCl pH 7.5, 100 mM NaCl, 100 µM EDTA, and 75 µM of the fusion inhibitor 3G (Sunet al., 2006, Singethanet al., 2010) using a peristaltic pump (BioRad) at a flow rate of 0.7 ml/min. The supernatant was added to the column, which was then rinsed with 20 ml of buffer A (same flow rates). Finally, three subsequent elutions were performed by adding 1 ml of buffer B (HA peptide (Pierce™) reconstituted to 1 mg/ml in buffer A) to the resin. All purification steps were performed at 4 °C.

### 2.2 Cryo-EM grid preparation, data acquisition and movie processing

Purified CDV solF was diluted to 230 µg/ml with 20 mM Tris-HCl pH 7.5, 100 mM NaCl, 100 µM EDTA and 75 µM of the fusion inhibitor 3G. 2.5 µl were applied to glow-discharged (10 mA, 120 s, 0.25 mbar) holey carbon grids (Cu R2/1, 200-mesh, Quantifoil), blotted for 4.5 s and then plunge-frozen in liquid ethane using a Vitrobot Mark IV apparatus (Thermo Fisher Scientific) operated at approximately 100% humidity and cooled to 4 °C. The grids were stored in liquid nitrogen until further use.

Cryo-EM images were recorded using SerialEM (Mastronarde, 2005) at the C-CINA (Center for Cellular Imaging and NanoAnalytics) in Basel, Switzerland on a FEI Polara microscope (Thermo Fisher Scientific) operated at 300 kV and equipped with a K2 Summit direct electron detector (Gatan). Movies of 48 frames were recorded in super-resolution counting mode at a magnification of 35,000× (pixel size 0.5105 Å) with a dose of 1.516 e^-^/Å^2^/frame, resulting in a total accumulated dose on the specimen level of approximately 72.8 e^-^/Å^2^ per exposure.

**Fig. 1:**
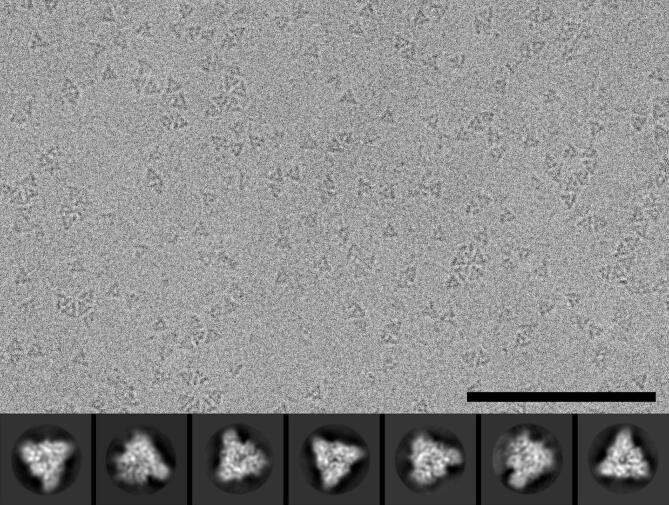
Cryo-EM of purified CDV solF protein. A representative electron micrograph (defocus −2.0 µm) of vitrified CDV solF particles (*top*) and 2D classes in different orientations (*bottom*) are displayed. The scale bar represents 100 nm and the frame size of the 2D classes in the gallery is 16 nm.

The Focus program (Biyani et al., 2017) was used to process the acquired movies during the imaging session with the following steps: Gain reference application by IMOD (Kremer et al., 1996), twofold binning by FREALIGN (Grigorieff, 2007), motion-correction and dose-weighting by MotionCor2 (Zheng et al., 2017), and contrast transfer function (CTF) estimation by CTFFIND4 (Rohou and Grigorieff, 2015). Images displaying high drift, ice-contaminations or a resolution worse than 8 Å according to the CTF estimation were excluded from further analysis, resulting in a total of 1,604 aligned movies (see Fig. 1 for a representative electron micrograph).

### 2.3 Image processing, model building and refinement

**Fig. 2:**
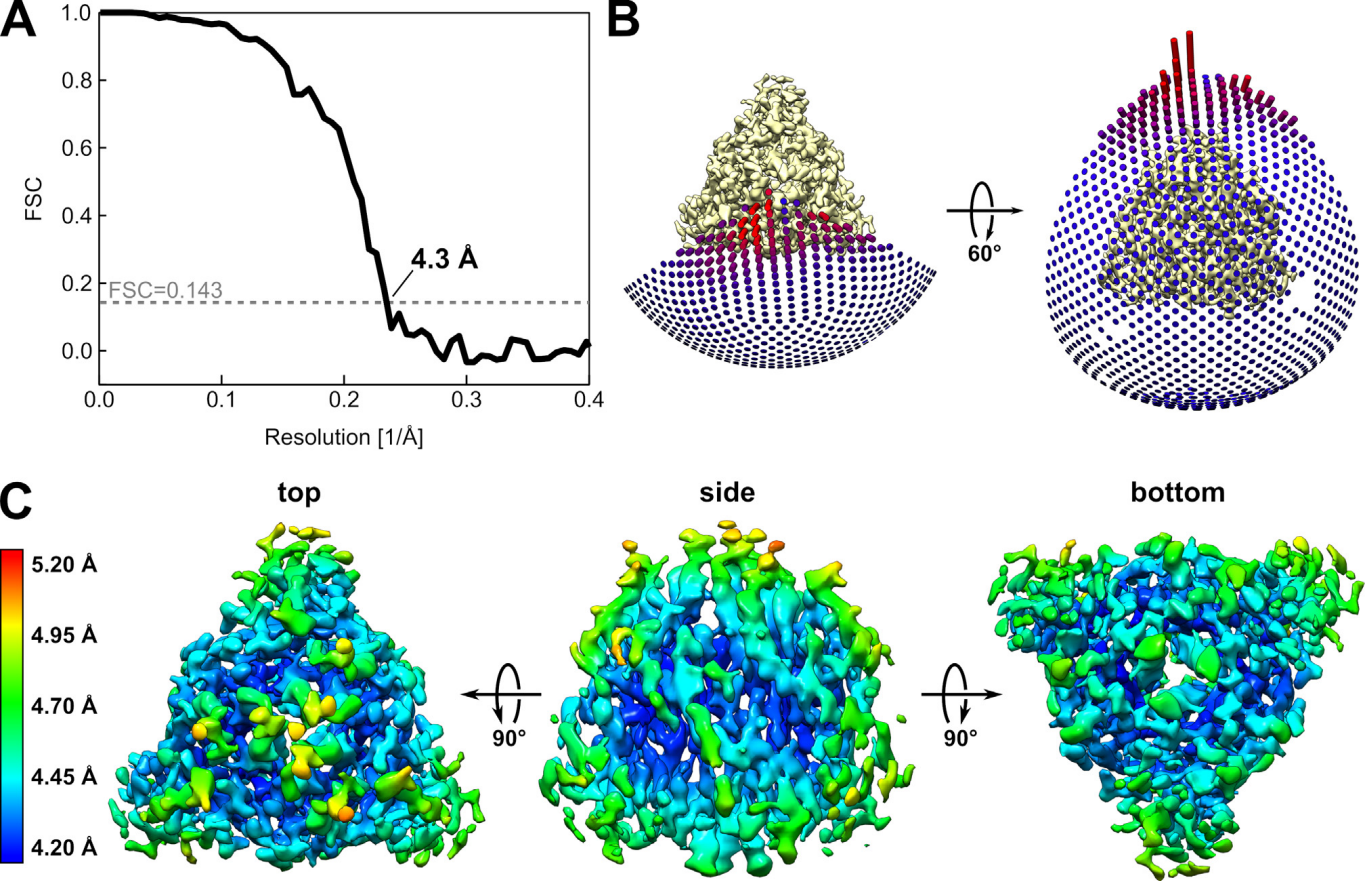
Cryo-EM data analysis of CDV solF protein. (A) The plot shows the Fourier shell correlation (FSC) curve of the final calculated density map. The reported resolution is based on the FSC = 0.143 criterion. (B) Angular distribution plots of particles included in the final 3D reconstruction viewed from two different angles. The height of the cylinder is proportional to the number of particles for the corresponding view. (C) The final 3D reconstruction of CDV solF is shown in three different views (top, side and bottom) and colored according to the local resolutions estimated by ResMap (Kucukelbir et al., 2014).

All image processing steps were performed with RELION-3 (Zivanov et al., 2018). An initial particle set was picked without templates using the auto-picking procedure based on a Laplacian-of-Gaussian filter and then 2D classified. The best 2D classes were used for a template based auto-picking resulting in a dataset of 491,315 particles, which was cleaned through three rounds of 2D classification. The remaining 182,589 particles were used to generate a rotationally symmetric (C3) starting 3D map with the stochastic gradient descent algorithm of the RELION-3 software for 3D processing. After two rounds of 3D classification (with eight and three 3D classes), 115,248 particles were used for 3D refinement and resulted in a 4.7 Å map. With the in RELION-3 newly implemented Bayesian polishing (Zivanov et al., 2019), the map resolution could be improved to 4.4 Å. 3D refinement, modulation transfer function (MTF) correction and map sharpening were performed with RELION-3, and resulted in a final map with an overall resolution of 4.3 Å according to the Fourier shell correlation (FSC) curve between two independently refined half-maps at FSC = 0.143 (Fig. 2A). Local resolution estimation was performed with the RELION-3 wrapper for the ResMap algorithm (Kucukelbir et al., 2014).

Homology model of CDV solF was generated by SWISS-MODEL (Waterhouse et al., 2018) based on the biological unit of the MeV fusion protein ectodomain (MeV solF) X-ray structure (PDB-ID: 5YZC). The PHENIX Dock in map tool (Liebschner et al., 2019) was used for initial rigid-body fitting and further refinement was performed with PHENIX real-space refinement (Afonine et al., 2018). To improve the fit and geometry of the model, several iterations of manual adjustment in Coot (Emsley et al., 2010) and structure validation with the MolProbity web service (Chen et al., 2010) were carried out. Data collection, processing and refinement statistics can be found in Table 1. The original electron micrographs, the cryo-EM map and the protein coordinates were deposited in the Electron Microscopy Public Image Archive (EMPIAR-10366), in the Electron Microscopy Data Bank (EMD-10649) and in the Protein Data Bank (PDB-ID: 6XYE), respectively. All volume and structural representations were prepared using Chimera v1.12 (Pettersen et al., 2004) or PyMol v2.3 (The PyMol Molecular Graphics System, Schrödinger).

**Table 1:** Data collection and model validation statistics.

**Data Collection**	
Microscope	FEI Polara
Voltage (kV)	300
Camera	Gatan K2 Summit
Defocus range (µm)	0.9 to 3.4
No. of movies	1,604
Frames per movie	48
Total dose per movie (e^−^/Å^2^)	72.8
Pixel size (Å)	0.5105

**Reconstruction**	
Software	RELION-3
Symmetry	C3
Particles refined	115,248
Map Resolution (Å)*	4.3
Map sharpening B-factor (Å^2^)	−206

**Refinement and Validation**	
Software	Phenix 1.16–3546
Model composition	
Non-hydrogen atoms	9,834
Protein residues	1,293
MolProbity Score	1.88
All-atom clashscore	10.97
Rotamers	
Favored (%)	98.1
Outliers (%)	0
Ramachandran	
Favored (%)	95.3
Outliers (%)	0
RMS deviations	
Bond length (Å)	1.37
Bond angles (°)	0.01
Mean B-factors	
Protein (Å^2^)	87.7
CaBLAM outliers (%)	0.9
C_α_ Geometry outliers (%)	0
C_β_ Deviations > 0.25 Å (%)	0

*Resolution was determined by FSC between two half-maps using 0.143 as a cutoff.

## 3 Results and discussion

### 3.1 CDV F ectodomain expression and purification

For cryo-EM single particle reconstruction, the previously reported CDV solF construct (Ader et al., 2013) was expressed in HEK293 cells and purified by affinity chromatography (for details, see Materials and methods). In this construct, the transmembrane domain and the cytoplasmatic C-terminal part were replaced by a trimeric GCN3 motif (GCNt), which should stabilize the prefusion state. However, we have recently shown by negative-stain electron microscopy, that despite this prefusion-stabilizing GCNt trimerization motif, a considerable amount of solF spontaneously refolded into postfusion state (Kalbermatter et al., 2019). Therefore, to ensure a sample of predominantly prefusion state solF for cryo-EM single particle reconstruction, a high concentration (75 µM) of the fusion inhibitor 3G (Sunet al., 2006, Singethanet al., 2010) was present during protein expression and purification.

### 3.2 Structure determination and overall structure of CDV solF

Cryo-EM data of purified solF were acquired on a 300 kV FEI Polara electron microscope equipped with a direct electron detector. Electron micrographs displayed a homogenous distribution of triangular shaped particles (Fig. 1) corresponding to the prefusion state of CDV solF, because in the postfusion state CDV solF adopts a less compact and more elongated tadpole-like shape (Kalbermatter et al., 2019). Also after reference-free auto-picking and initial 2D-classification, no postfusion-like 2D classes appeared. This shows that the fusion inhibitor 3G efficiently stabilizes the prefusion state of CDV solF. The initially calculated 2D classes were used for a template-based auto-picking and the resulting particles were cleaned through several rounds of 2D classification before an initial 3D map was calculated (Materials and methods). After further removal of bad particles by rounds of 3D classification and improving the 3D map by particle polishing, 3D refinement and map sharpening, a final 3D map was calculated from 115,248 particles. Despite the preferred orientations of these particles (Fig. 2B), a 3D reconstruction with an overall resolution of 4.3 Å was obtained (Fig. 2A; Materials and methods). The local resolution analysis of the map showed that in the core of the map the resolution was better (i.e., <4.3 Å) than at the periphery (Fig. 2C).

**Fig. 3:**
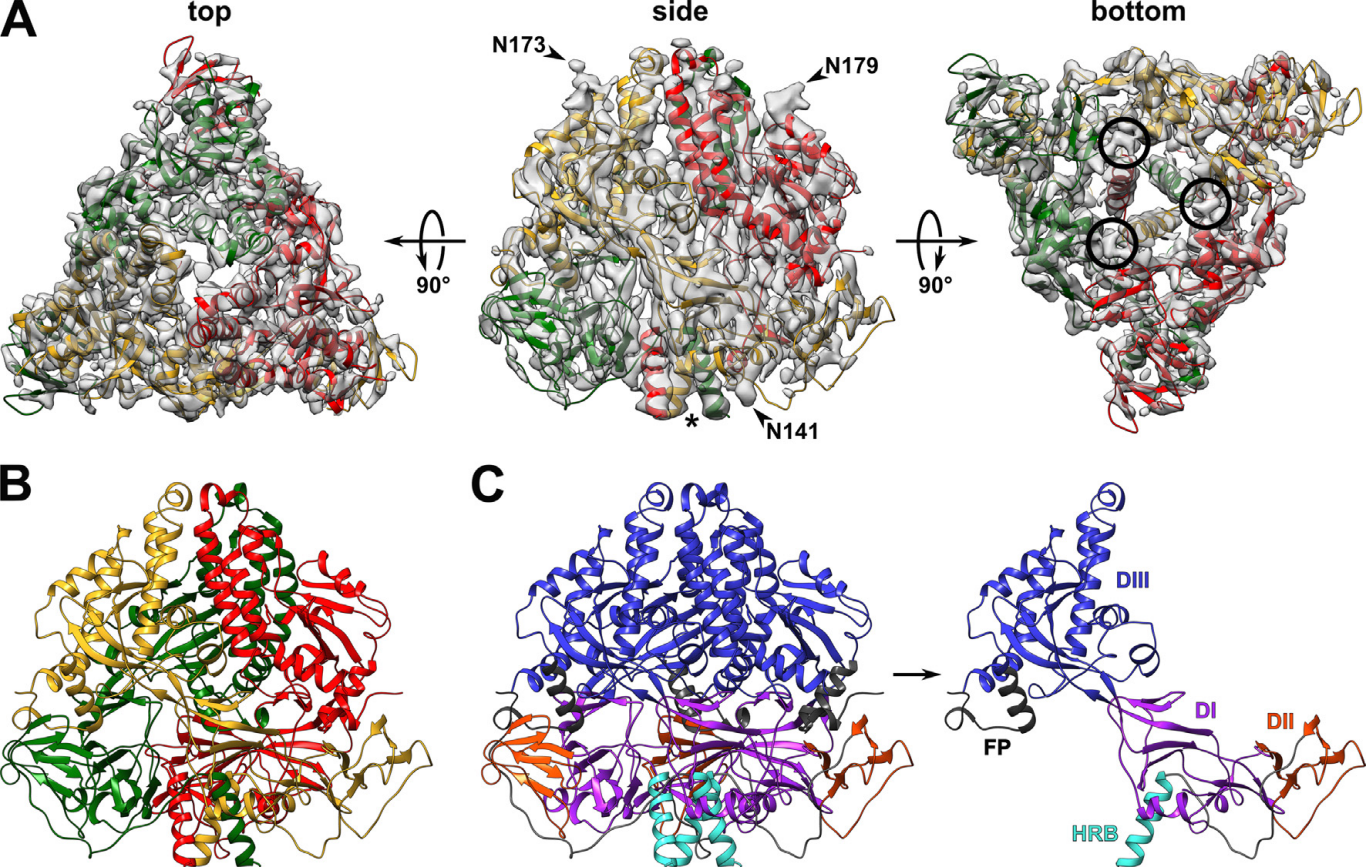
Cryo-EM map and model of prefusion CDV solF trimers. (A) Three different views of the prefusion CDV solF protein model and the cryo-EM map are shown. The three protomers of the model are differently colored (yellow, red and green) and displayed as a ribbon model. The cryo-EM map is visualized as a transparent volume. In the side view, the stalk region is indicated by an asterisk and the densities of the three N-linked glycosylation sites (N141, N173 and N179) are marked with arrowheads. In the bottom view, the locations of the three binding sites with the densities for the inhibitor 3G are highlighted with black circles. For more clarity, the views were slabbed. (B) The full CDV solF model is shown and viewed from the side. The trimeric nature of CDV F is indicated by three different colors for the three protomers (yellow, red and green). (C) On the left side, all the three protomers of CDV F are shown and colored according to the different structural domains as defined for the prefusion PIV5 F structure (Yin et al., 2006). On the right side, only one protomer is shown with the same coloring: HRB, cyan; Fusion Peptide (FP), black; DI, magenta; DII, orange; DIII, blue.

Since the sequence identity between the ectodomains of CDV F and MeV F is high (~72%), a homology model based on the X-ray structure of the MeV solF (Hashiguchi et al., 2018) was generated and fitted into the cryo-EM density map. The geometry of the structure and the fit into the map was improved manually and through several real space refinement runs. The final CDV solF model is shown in Fig. 3A and B, which displays the known “tree”-like fold of the prefusion state of morbillivirus F proteins (Plattet et al., 2016): The HRB domain of the three chains form a helix bundle, i.e., a short stalk, which supports a large globular head domain consisting of the three subdomains DI, DII and DIII (Fig. 3C). The DI and DII subdomains of the three monomers form the base of the head and are composed of mainly β-sheets, i.e., DI two short α-helices and three antiparallel β-sheets with different amounts of strands (i.e., two, three and four strands) and DII two three-stranded antiparallel β-sheets. The DIII subdomains consist of α-helix and β-sheet elements, and are located at the top of the head structure. One DIII subdomain consists of two long α-helices, five shorter α-helices with less than ten residues per helix and a five-stranded twisted antiparallel β-sheet. The fusion peptide (FP), which is built from mostly hydrophobic residues and is important for membrane fusion (Plattet et al., 2016), is buried between a DII and DIII subdomain of two different monomers.

The CDV F protein has three N-linked glycosylation sites. This is consistent with previous biochemical studies, which demonstrated those sites to be glycosylated (von Messling and Cattaneo, 2003). One N-linked glycosylation site (N173) has been shown to be important for proper protein folding, whereas the other two (N141 and N179) play a supporting role in the fusion function (von Messling and Cattaneo, 2003). In the CDV solF structure, N173 and N179 are located at the top of the head domain in the DIII subdomain, and N141 is located at the bottom close to the HRB region in the DI subdomain. Interestingly, for all three N-linked glycosylation sites, additional densities were found in the cryo-EM map (arrowheads in the side view of Fig. 3A), which would correspond to glycans. This indicates that the recombinantly expressed CDV solF protein is glycosylated.

### 3.3 Inhibitor binding pocket location and description

In the MeV solF crystal structure it has been shown that AS-48 binds to a hydrophobic pocket located in the region connecting the head and the stalk of the MeV F trimer with a stoichiometry of three inhibitors per MeV F trimer (Hashiguchi et al., 2018). Since 3G is a derivative of AS-48 (Plemper et al., 2005) and the compounds exhibit IC_50_s for membrane fusion in the single digit micromolar range against CDV and MeV (Singethan et al., 2010), it is expected that both inhibitors bind to the same site. Indeed, the cryo-EM map of CDV solF showed additional densities (circles in bottom view of Fig. 3A and Supplementary Fig. 1) in the region of the AS-48 binding site identified in the MeV solF crystal structure (Hashiguchi et al., 2018).

**Fig. 4:**
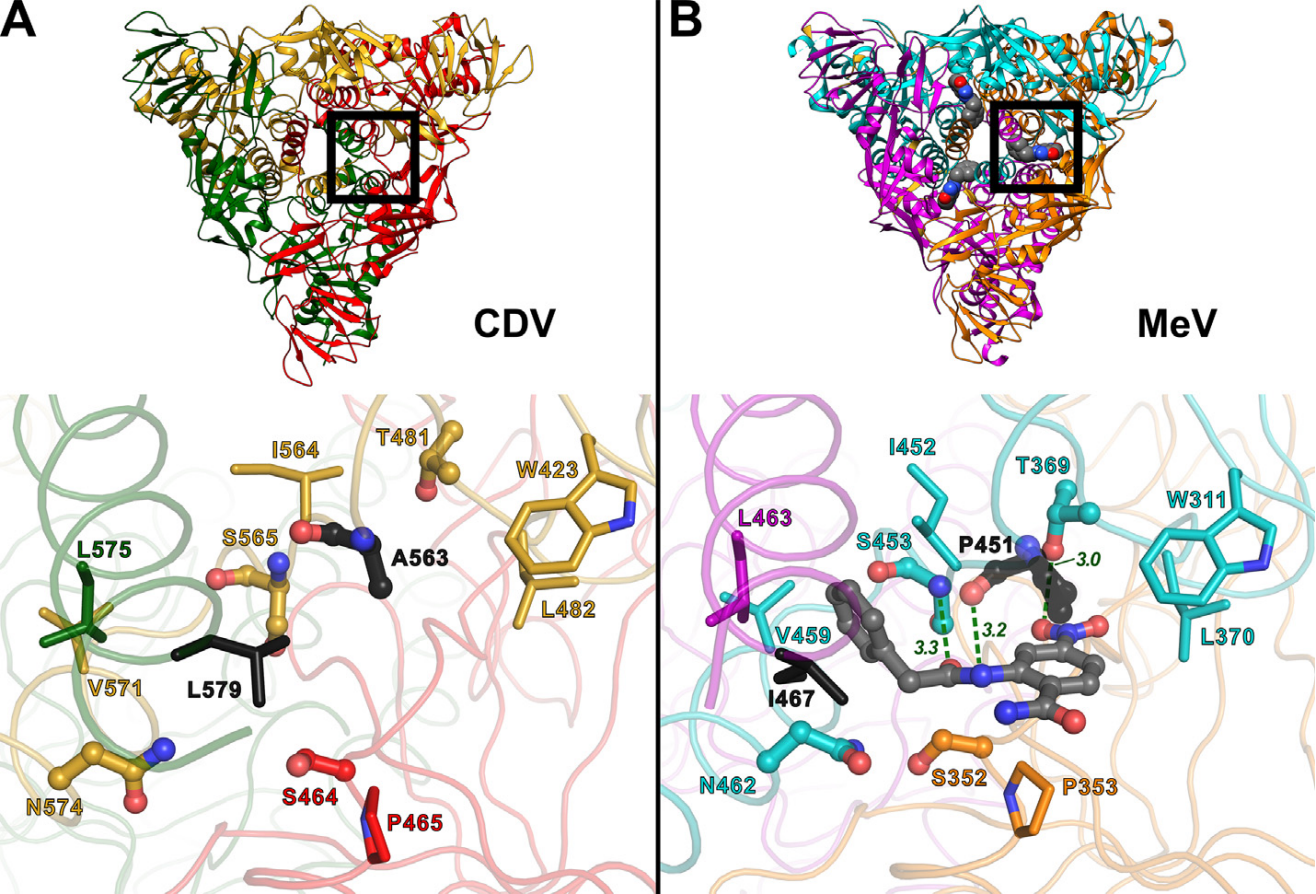
Comparison of the inhibitor binding pocket of CDV solF and MeV solF. Bottom views of the prefusion CDV solF (A) or MeV solF (PDB entry 5YZC; (Hashiguchi et al., 2018)) structures (B) are displayed (*top*). In the models, the three protomers are differently colored: (A) yellow, red and green; (B) cyan, orange and magenta. A magnified view of the CDV solF binding pocket (A) and the same view of the MeV solF binding pocket with bound AS-48 (B) are shown (*bottom*). The side chains of the amino acids within a distance of 4 Å from the AS-48 molecule in MeV solF and the corresponding amino acids in CDV solF are displayed. The hydrophobic amino acid residues are displayed as sticks and the amino acid residues in hydrogen bonding distance to AS-48 in MeV solF (B) (and the corresponding amino acids in CDV solF (A)) are displayed as ball-and-sticks. The hydrogen bonds to AS-48 are indicated with broken lines and the distances are given in Å. Amino acids that are different between CDV and MeV are colored in black. The protomers are colored as in the models in the top panel.

All three protomers of the CDV solF trimer are involved in one single putative inhibitor binding pocket. Despite the similar fold and the high sequence identity (~72%) between the MeV F and CDV F ectodomains, two amino acids involved in inhibitor binding are different between the two morbilliviruses. At position 467 in MeV F, CDV F has also a hydrophobic amino acid, i.e., a leucine (L579) instead of an isoleucine (Fig. 4). The more interesting difference is the alanine at position 563 in CDV F instead of a proline (P451) in MeV, because proline has an exceptional conformational rigidity compared to alanine (black amino acids displayed as ball-and-stick in Fig. 4). Indeed, the backbone carbonyl oxygen of A563 in CDV solF is slightly turned away from the center of the putative inhibitor binding pocket compared to the backbone carbonyl oxygen of P451 in MeV solF (Fig. 4). This backbone carbonyl oxygen is in hydrogen bonding distance to the amide nitrogen of AS-48, which suggest an important role of P451 in the inhibitor binding mechanism. Despite the hydrophobic nature of the fusion inhibitor binding pocket, AS-48 in MeV solF can form three hydrogen bonds with the surrounding amino acids: (i) the above mentioned hydrogen bond between the backbone carbonyl oxygen of P451 and the amide nitrogen of AS-48; (ii) the protein backbone nitrogen of S453 and the carbonyl oxygen of AS-48, and (iii) the side chain hydroxyl group of T369 and the nitro-group of AS-48 (Fig. 4B).

The additional densities observed in the CDV solF 3D map (circles in bottom view of Fig. 3A and Supplementary Fig. 1) at sites corresponding to the AS-48 inhibitor binding pockets in the MeV solF structure (Hashiguchi et al., 2018) together with our previous observation that 3G stabilizes the prefusion state in our recombinant protein (Kalbermatter et al., 2019) led us to the conclusion that 3G binds to CDV solF also with a stoichiometry of three molecules per trimer. However, because of the moderate overall resolution of the calculated cryo-EM map, no reliable determination of the mode of binding of the fusion inhibitor could be drawn. We hence decided to exclude 3G molecules from the CDV solF model.

In summary, we have provided the cryo-EM structure of CDV solF prefusion state at 4.3 Å resolution. The overall solF architecture is similar to related paramyxovirus fusion proteins in their prefusion conformations such as MeV. Comparison of the fusion inhibitor binding sites of solF from CDV and MeV revealed minor differences, which could allow for the design of improved versions of inhibitors common to both morbillivirus fusion machineries. Importantly, the CDV solF prefusion structure now offers new opportunities to further engineer the protein and achieve high-quality immunogens for next-generation vaccine design and development of desired antiviral drugs.

## Author contributions

D.F., P.P. and R.R. conceived and designed the experiments. D.K., N.S., F.M.G. and M.W. performed the experiments. D.K. analyzed the cryo-EM data and solved the structure. D.K., P.P. and D.F. wrote the manuscript. All authors contributed to manuscript revision and approved the final version.

## Declaration of Competing Interest

The authors declare that they have no known competing financial interests or personal relationships that could have appeared to influence the work reported in this paper.
