# Supplementary material for: Cryo-EM structure of the prefusion state of canine distemper virus fusion protein ectodomain
Source: J Struct Biol X. 2020 Feb 29;4:100021. doi: 10.1016/j.yjsbx.2020.100021 (PMC7337061; doi:10.1016/j.yjsbx.2020.100021)
Supplement: Supplementary data 1 [file mmc1.doc]

**SUPPLEMENTARY DATA**

# Cryo-EM structure of the prefusion state of

# canine distemper virus fusion protein ectodomain

David Kalbermatter^a^, Neeta Shrestha^b,c^, Flavio M. Gall^d^, Marianne Wyss^b,c^, Rainer Riedl^d^, Philippe Plattet^b,c^, Dimitrios Fotiadis^a^

^a^ Institute of Biochemistry and Molecular Medicine, University of Bern, Bern, Switzerland

^b^ Division of Experimental and Clinical Research, Vetsuisse Faculty, University of Bern, Bern, Switzerland

^c^ Division of Neurological Sciences, Vetsuisse Faculty, University of Bern, Bern, Switzerland

^d^ Center of Organic and Medicinal Chemistry, Institute of Chemistry and Biotechnology, Zurich University of Applied Sciences ZHAW, Wädenswil, Switzerland


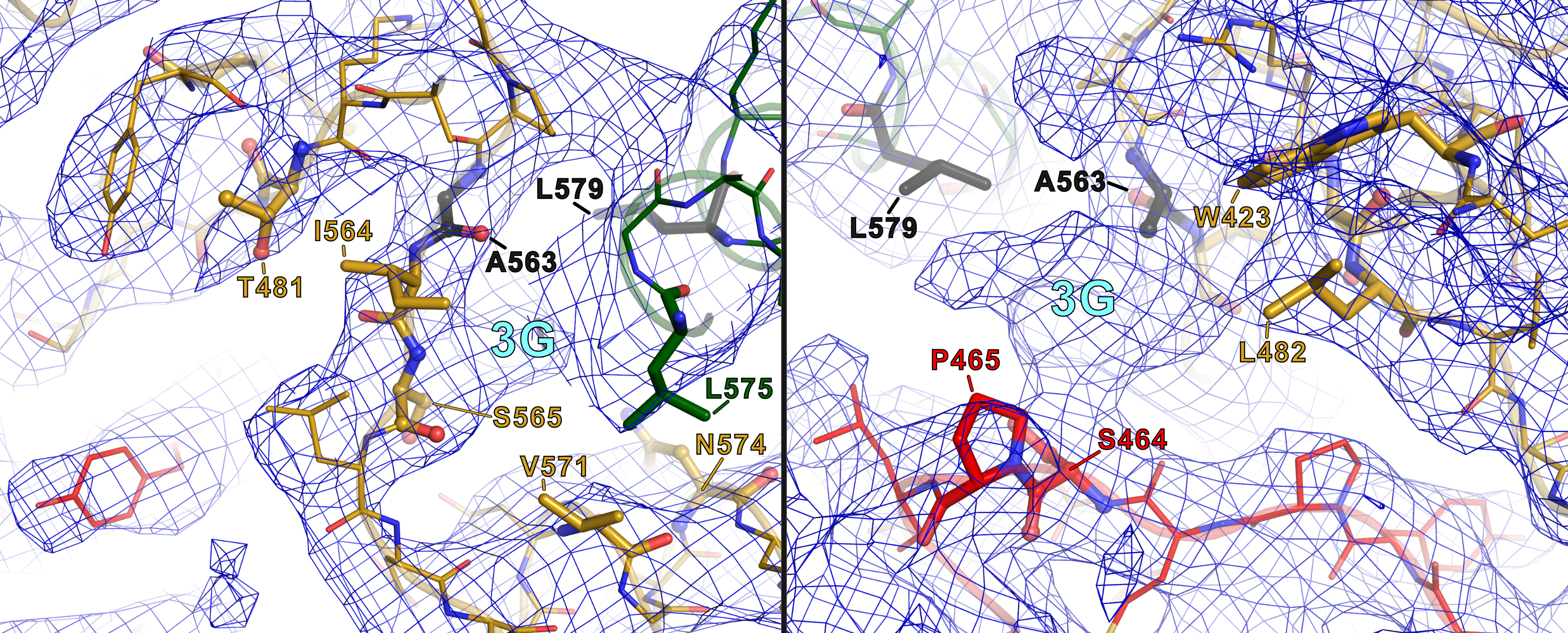


**Supplementary Fig. 1.** Views into the 3G inhibitor binding pocket of CDV solF. The cryo-EM density map is shown in blue and contoured at 1.5 σ. The representation and coloring of the model is the same as in Fig. 4: The three protomers of the model are colored in yellow, red and green. Amino acids that are different between CDV and MeV are colored in black. Hydrophobic amino acid residues in the inhibitor binding pocket are displayed as sticks and the analogous amino acid residues in CDV solF, which in MeV solF are in hydrogen bonding distance to AS-48 are displayed as ball-and-stick. The additional density for the inhibitor 3G is indicated in both panels (in cyan font color). Amino acid side chains for which the density was not well-defined, because of the moderate resolution, rotamers of high probability were chosen. The views were slabbed for better appearance.
